# Lifespan extension and delay of age-related functional decline caused by *Rhodiola rosea* depends on dietary macronutrient balance

**DOI:** 10.1186/2046-2395-2-5

**Published:** 2013-04-02

**Authors:** Dmytro V Gospodaryov, Ihor S Yurkevych, Mahtab Jafari, Volodymyr I Lushchak, Oleh V Lushchak

**Affiliations:** 1 Department of Biochemistry and Biotechnology, Vassyl Stefanyk Precarpathian National University, Ivano-Frankivsk 76025, Ukraine; 2 Department of Pharmaceutical Sciences, University of California, Irvine, CA 92697, USA; 3 Department of Zoology, Stockholm University, S-10691 Stockholm, Sweden

**Keywords:** Adaptogen, Diet, Fecundity, Fruit fly, Longevity, Macronutrient balance, *Rhodiola rosea*

## Abstract

**Background:**

This study was conducted to evaluate the effects of rhizome powder from the herb *Rhodiola rosea*, a traditional Western Ukraine medicinal adaptogen, on lifespan and age-related physiological functions of the fruit fly *Drosophila melanogaster*.

**Results:**

Flies fed food supplemented with 5.0 mg/ml and 10.0 mg/ml of *R. rosea* rhizome powder had a 14% to 17% higher median lifespan, whereas at 30.0 mg/ml lifespan was decreased by 9% to 12%. The preparation did not decrease fly fecundity.

The effect of *R. rosea* supplement on lifespan was dependent on diet composition. Lifespan extension by 15% to 21% was observed only for diets with protein-to-carbohydrate ratios less than 1. Lifespan extension was also dependent on total concentration of macronutrients. Thus, for the diet with 15% yeast and 15% sucrose there was no lifespan extension, while for the diet with protein-to-carbohydrate ratio 20:1 *R. rosea* decreased lifespan by about 10%.

Flies fed *Rhodiola* preparation were physically more active, less sensitive to the redox-cycling compound menadione and had a longer time of heat coma onset compared with controls. Positive effects of *Rhodiola* rhizome on stress resistance and locomotor activity were highest at the ‘middle age’.

**Conclusions:**

The present data show that long-term food supplementation with *R. rosea* rhizome not only increases *D. melanogaster* lifespan, but also delays age-related decline of physical activity and increases stress resistance, what depends on protein-to-carbohydrate ratio of the diet.

## Background

Over the past few decades, the fruit fly *Drosophila melanogaster*, nematode *Caenorhabditis elegans*, and budding yeast *Saccharomyces cerevisiae* have been extensively used for lifespan studies because of their relatively short lifecycles and, especially, the ease of producing knockouts of specific genes. In addition to genetic manipulations, these organisms are also used in the search for anti-aging medicinal preparations. For instance, studies on *S. cerevisiae* disclosed anti-aging properties of resveratrol, a plant-derived compound, well established by its presence in some types of wine. *Drosophila* has also been checked for anti-aging properties of resveratrol [1,2], as well as 4-phenylbutyrate [3], caffeine [4], curcumin [5], statin [6], *Rhodiola rosea*[7,8] and *Rosa damascena*[9], blueberry extract [10] and many other preparations. Changes in diet composition, namely dietary and caloric restriction, were also found to extend fruit fly lifespan [11]. Several molecular mechanisms have been proposed for dietary or drug-mediated longevity enhancement. In particular, FOXO (forkhead box O), TOR (target of rapamycin) and AMPK (AMP-activated protein kinase) signaling pathways are believed to be involved in lifespan-prolonging effects of many treatments, as determined by experiments conducted on fruit fly models [12,13]. These pathways are now extensively investigated in many aspects, and many interconnections between them have already been reported. One of the common features of all these signaling pathways is their relation to the stress resistance of organisms [12,14].

Notably, in many cases, an extended lifespan is accompanied by an increased stress resistance of survivors who have consumed food supplemented with an anti-aging medication. Moreover, one of these life-prolonging preparations, *Rhodiola rosea*, is a well-known adaptogenic herb. This plant is widely used in folk medicine among Ukrainians living in the Carpathian Mountains, as well as among people in other regions of Eurasia, including Finland, Russia, and China. Preparations from rhizome-like roots of this plant are shown to have cardioprotective, antidepressant, anticancer, antihyperglycemic, antinarcotic, and other beneficial activities [15,16]. Multiple studies reported that *R. rosea* extract can enhance resistance to heat stress, heavy metals, and redox-cycling agents [17]. Experiments on the adaptogenic properties of *R. rosea* have been conducted in animal models, including rats [15,18], mollusks [17], and worms [19]. Recent studies in *Drosophila melanogaster* have shown that *R. rosea* can also be used as an anti-aging pharmacological agent [7]. The intriguing relationship between aging and stress resistance is increasingly mentioned in contemporary gerontology [20]. Moreover, it was shown that many adaptogens have anti-aging properties; conversely, many anti-aging preparations were found to increase adaptive capabilities [21]. It has also been hypothesized that stressed plants can synthesize stress-signaling molecules, which increase stress resistance of herbivorous species [22].

In this study, we pursued several goals. Particularly, we wished to reproduce and confirm previous results [7,8] independently, running experiments in Ukraine on a fruit fly line, caught from nature, and using freshly prepared unprocessed *R. rosea* rhizome. In some cases, especially for the newly discovered preparations, repeated tests in different laboratories are thought to be useful prior to the search for a molecular mechanism [23]. We also tried to validate adaptogenic properties of our rhizome preparations by checking the resistance of flies fed *R. rosea* to potential oxidative stress, exerted by menadione, and heat stress. Along with mobility and fecundity, stress resistance can also be examined as an estimate of ‘life quality’. It is noteworthy that not all life-extending medications can improve healthspan [24], and this may not be acceptable in the medical sense. Thus, checking healthspan indices would be important for recognition of an anti-aging remedy with minimum negative side effects. Here, we present data on the life-long stress resistance of fruit flies fed a diet supplemented with *R. rosea* rhizome powder.

Our last goal was to define optimal dietary conditions for the anti-aging effect of *R. rosea*. This effect can be modulated by different factors, and diet is thought to be one of the most critical. It is well established that diet itself can prolong or shorten lifespan [25]. Recent studies have shown that the median lifespan of a population depends not only on the total caloric value of the diet but also on the dietary composition [26-28], especially the protein-to-carbohydrate ratio [29]. If diet modulates the effect of anti-aging pharmacological intervention, the maximum life-prolonging effect may be seen only for some certain dietary conditions, whereas others might not be so favorable. The same is true for health-promoting effects. The dietary response could also provide some implications for the primary molecular targets of *R. rosea* bioactive compounds, among which salidroside, rosavins, and p-tyrosol are the most studied. In this work, we show how dietary composition may affect life extension and food intake with *R. rosea* supplement.

## Results and discussion

### *Rhodiola* supplementation increases median lifespan

Previously, it was found that *R. rosea* increased lifespan in fruit flies [7,8]. These studies were performed at the University of California in Irvine (UCI) using *R. rosea* powder manufactured and processed in China, and a *Drosophila melanogaster* line, caught in nature and kept for a long time at the laboratory conditions. In Ukraine, *Rhodiola rosea*, called ‘golden root’, is a famous medicinal herb known to increase working capacity, stamina, and health in general. The plant grows predominantly in mountainous areas around 1000 m above mean sea level. In the Carpathian Mountains, *R. rosea* is relatively accessible, and the fresh roots can be collected. Virtually every year, research teams from medical universities in the West Ukraine publish their results on multiple health benefits provided by administration of *R. rosea* extract in local journals. Here we tried to reproduce experiments conducted previously using freshly prepared roots of *R. rosea* and a wild *D. melanogaster* line collected in western Ukraine.

**Figure 1:**
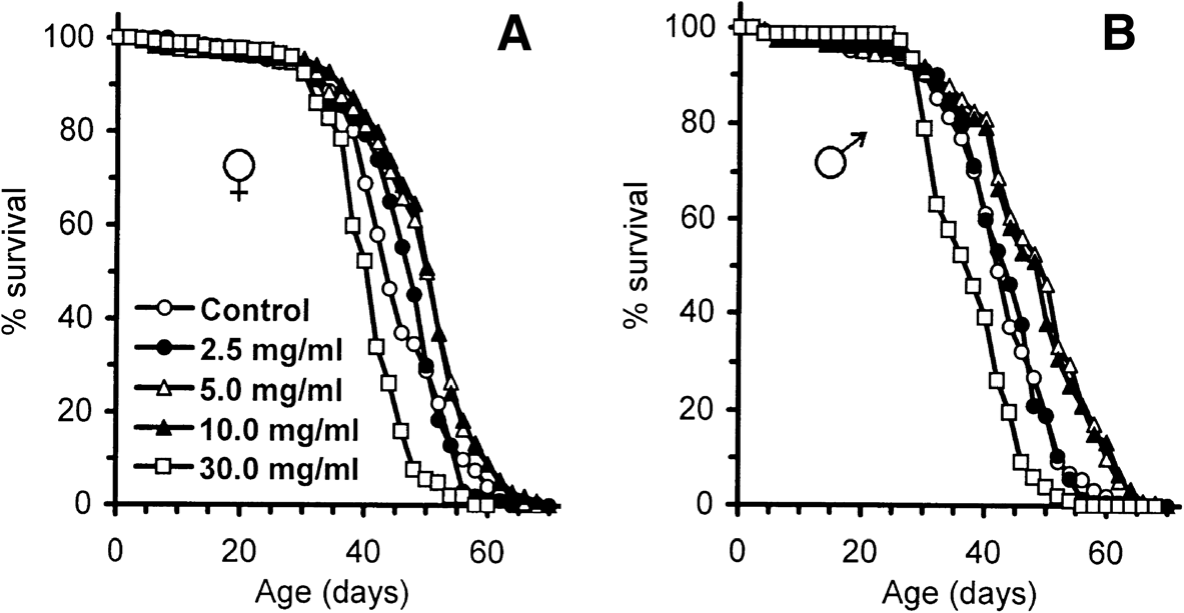
**Survivorship of female (A) and male (B) flies fed with different concentrations of *R. rosea *rhizome.** Results are representative of three separate experiments with about 100 to 400 flies per sex, per diet.

We have found that supplementation of fruit fly food, containing 5% yeast and 5% sucrose, (approximately 6.25% carbohydrate and 2.25% protein) with 5.0 mg/ml and 10.0 mg/ml of *R. rosea* rhizome extended the median lifespan of flies of both sexes by 14% to 17% (Figure 1, Table 1). The same concentrations of *R. rosea* rhizome increased maximum lifespan of the females by between 3% and 6%. No significant effects on maximum lifespan were observed in males fed with food supplemented by *R. rosea* in mentioned concentrations. Notably, females and males fed diet supplemented with 2.5 mg/ml and 30.0 mg/ml rhizome had, respectively, a 6% and 15% lower maximum lifespan, compared with the control. Moreover, 30.0 mg/ml of *R. rosea* decreased median lifespan by 9% to 12% (Table 1, Figure 1), demonstrating possible toxic effects of the rhizome at the higher concentrations.

**Table 1:** **Lifespan trials of the control flies and flies fed diets supplemented with *R. rosea *** **rhizome**

**Sex**	***R. rosea *** **rhizome concentration (mg/ml)**	**Median lifespan (days)**	**Degree of median lifespan extension or reduction (%)**	**Maximum lifespan (days)**
Females				
	0 (control)	44		61.3 ± 0.5
	2.5	47	+6.8	59.5 ± 0.7
	5.0	50*	+13.6	63.4 ± 0.6**
	10.0	50*	+13.6	63.7 ± 0.5**
	30.0	40*	−9.1	53.8 ± 0.4**
Males				
	0 (control)	42		60.3 ± 0.6
	2.5	43	+2.4	56.7 ± 0.6
	5.0	49*	+16.7	64.3 ± 0.5**
	10.0	48*	+14.3	63.9 ± 0.4**
	30.0	37*	−11.9	50.4 ± 0.8**

*Significantly different (*P* < 0.05) from control group as evaluated by Wilcoxon rank sum test. **Significantly different in comparison with control according two-way analysis of variance for normal data with Bonferroni correction.

In previous studies, flies were fed diets containing yeast paste mixed with bioactive compounds from *R. rosea*, as either rhizome powder [7] or standardized rhizome extract [8]. The lifespan-extending concentration of *R. rosea* powder, which did not lead to a significant decrease in fecundity, was 60 mg/ml powder [7]. When the extract was used, its active concentration was 25 mg of the extract per 1 ml yeast paste; however, 125 mg/ml extract exhibited a maximum effect. In these experiments, amounts of *R. rosea* powder mixed into fly food were several times smaller, while the highest concentration, 30.0 mg/ml, shortened lifespan. Nevertheless, it is now possible to say that *R. rosea* definitely increases the median lifespan of fruit fly cohorts, regardless of the preparation type, supplementation method, basic diet, or fruit fly line. The maximum increase in mean lifespan, shown in previous studies, was around 30%. In this study, an increase of up to 17% was obtained for the diet with 5% and 5% sucrose, with 10.0 mg of *R. rosea* rhizome powder per 1 ml of food.

The differences in the lifespan-prolonging effects obtained in previous studies and this one can be related to differences in the method of *R. rosea* supplementation and diet. In particular, the research team in UCI used banana-molasses food, which contains a different set of vitamins and other essential micronutrients than the plain yeast-and-sucrose food used in the current experiments. It is possible that *R. rosea* might prolong lifespan even further in combination with vitamins and other bioactive substances. Moreover, consumption of yeast paste by fruit flies could be less than consumption of ordinary food containing carbohydrates. This could also be the cause of the reductions in fruit fly fecundity with *R. rosea* preparation found in previous studies. It is known that ingestion of yeast influences the amount of eggs laid by female fruit flies [27,30]. When yeast and *R. rosea* preparation are combined in one supplement, the preparation might affect consumption of the whole supplement, and even at the level of food choice. In this study, we have suggested that *R. rosea* might influence food consumption and in this way regulate lifespan.

### *Rhodiola* decreases food consumption in fruit flies

**Figure 2:**
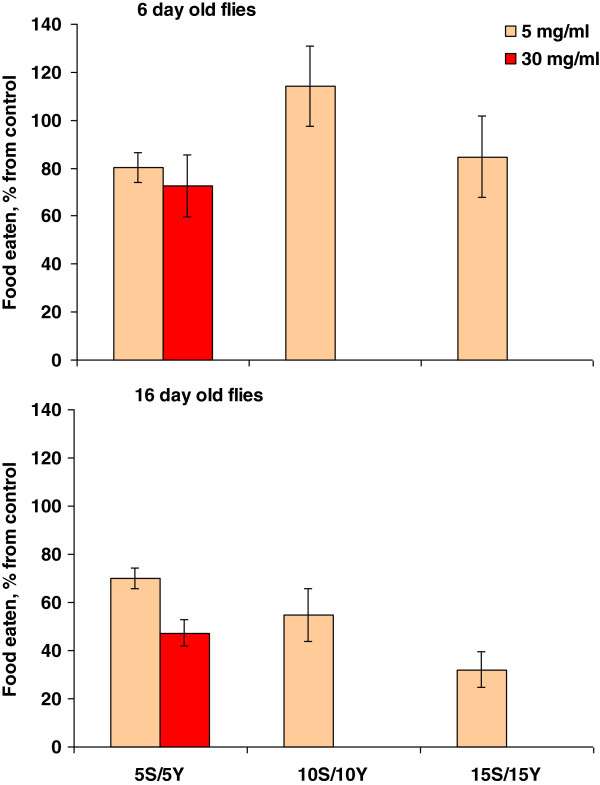
**Effect of *R. rosea *supplement on food consumption rate.** The difference between 6- and 16-day-old females is significant with *P* < 0.05 on 5S:5Y and 15S:15Y diets as evaluated by Student’s *t* test.

Food intake was assessed in 6- and 16-day-old females by the amount of food dye, erioglaucine, ingested within 15 min intervals. Six-day-old flies kept on *Rhodiola*-supplemented food did not show significant difference in food consumption as compared with control flies (Figure 2), while 16-day-old individuals fed food supplemented by 5.0 mg/ml *Rhodiola* rhizome consumed 1.4-, 1.8-, and 3.1-fold less food than controls on diets containing 5%, 10%, and 15% yeast and sucrose, respectively. However, addition of 30.0 mg/ml of *Rhodiola* supplement led to a halving in ingestion of the food containing 5% yeast and 5% sucrose. Alternatively, feeding was quantified by counting the flies sitting on the food surface directly, as described in [31]. The percentage of flies on the surface of the media with their proboscis extended and touching the food was significantly lower when food was supplemented with 5.0 mg/ml *Rhodiola* (Additional file 1: Figure S1). Thus, a reduced intake of yeast paste in previous studies could be the reason for reduced fecundity, despite it having been shown previously that the influence of *R. rosea* on yeast consumption is not significant [7].

It was shown that feeding behavior of *D. melanogaster* is regulated by many mechanisms, including the TOR pathway and its target S6 kinase, the insulin signaling pathway, and developmental hormones. It was recently shown that feeding behavior of *D. melanogaster* larvae could be regulated by S6 kinase, the downstream target of TOR and phosphoinositide-dependent kinases [32]. Larvae with up-regulated S6K consumed less food. However, a decrease in food consumption, conferred by *R. rosea* preparation, implies activation of S6 kinase [32], and, hence, activated TOR kinase. In turn, S6 kinase may inhibit the insulin signaling pathway by phosphorylation of insulin receptor substrate, thus promoting activity of transcriptional factor FOXO (forkhead box O) [33]. It was found that DAF-16, the FOXO homolog in *C. elegans*, might be involved in the life-prolonging effect of *R. rosea* preparations [19]. There are also other regulators of food intake; one of these is the juvenile hormone-binding protein Takeout [34]. It is known that Takeout mutation results in the increased food ingestion. However, Takeout has been shown to regulate feeding time, rather than ingestion rate [31].

Food intake in *D. melanogaster* is also regulated by biogenic amines, particularly by serotonin or octopamine [35]. Increased serotonin concentration is considered a satiety signal [36] and inhibits food consumption in insects [37]. It was also suggested that serotonin and octopamine might regulate insulin secretion by insulin-producing cells in *Drosophila* brain [36,38], thus potentially influencing the insulin signaling pathway. This explanation seems plausible, since *R. rosea* rhizome contains many compounds, such as kaempferol [15], found as inhibitors of monoamine oxidase, an enzyme catalyzing oxidation of biogenic amines [39]. Moreover, it was shown that *R. rosea* preparations are able to inhibit monoamine oxidase [40]. On the other hand, some monoamine oxidase inhibitors, as well as some monoamines themselves [41], were shown to extend lifespan [42].

Notably, our data have shown that *R. rosea* does not extend lifespan as much as the *mth* or *chico* mutations [43,44], or other compounds, for example, 4-phenylbutyrate [3]. In this case, it would be interesting to know which age stratum of individuals would benefit most from the *R. rosea* treatment. Pursuing the latter goal, we have analyzed Gompertz equation parameters as the next step.

### Consumption of *Rhodiola* preparation causes changes in age-independent and age-dependent mortalities

A model described by the Gompertz equation is a simple method to analyze survival data. This equation is

$$\mu \left(t\right)=Ae^{\mathrm{\alpha t}},$$

where *μ*(*t*) is the age-specific mortality rate, *t* is the age, *A* is the age-independent parameter and *α* is the age-dependent parameter. The higher *A* and *α*, the higher will be the mortality rate and, hence, the shorter the median and maximum lifespans. Age-independent mortality is thought to be caused by environmental factors or by genes responsible for early-age death [45].

**Figure 3:**
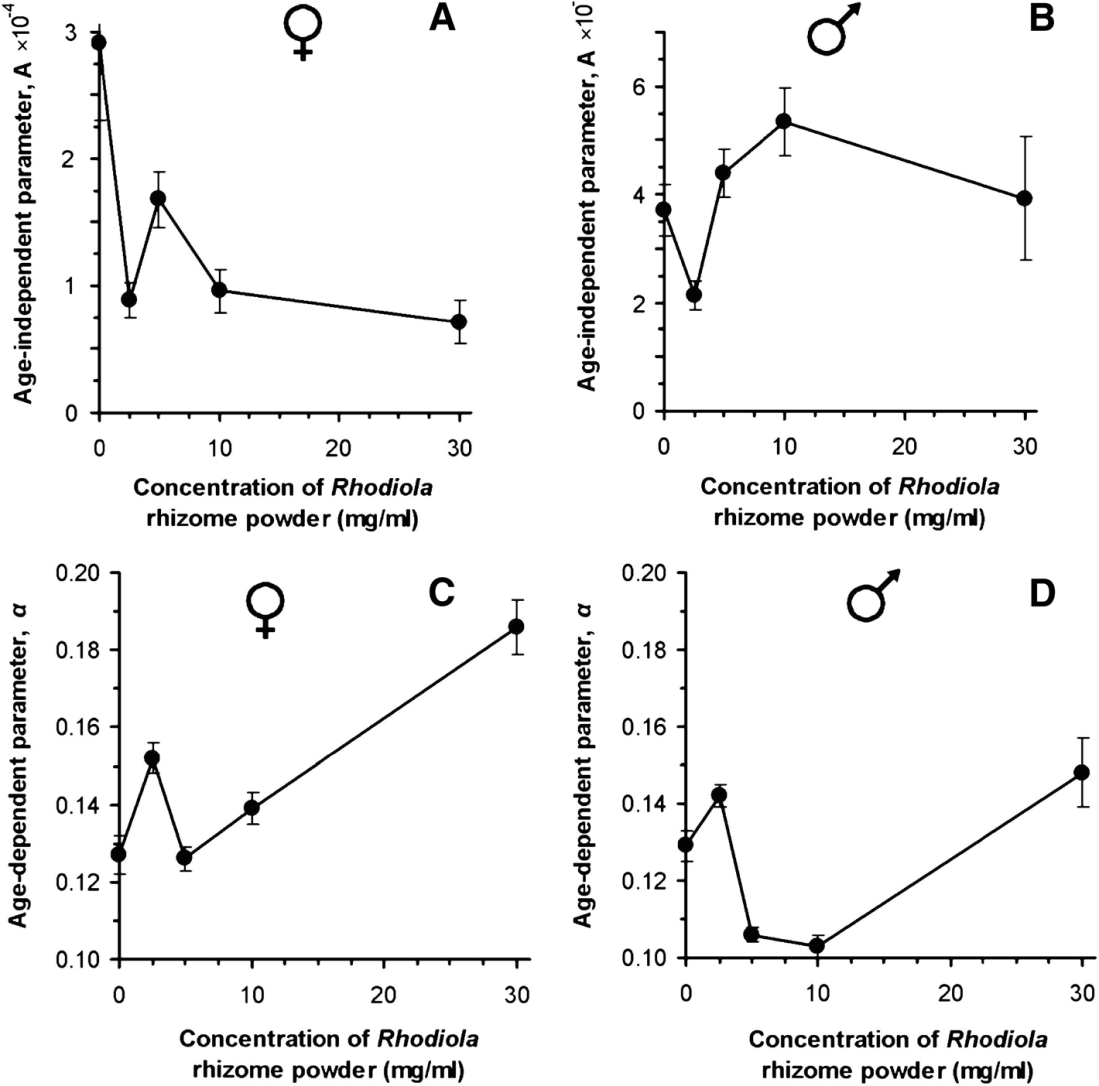
**Estimates of Gompertz equation parameters for the fly cohorts fed diets with different concentrations of *R. rosea *rhizome (A and C – A and *α*, respectively, for female cohorts, B and D – for male ones).** Error bars show the standard error of the parameter. All fit values were reliable (P< 0.05 by Student’s *t*-test).

We found that *R. rosea* lowered age-independent mortality in females, but slightly increased it in males (Figure 3). Moreover, males had higher *A* estimates than females, regardless of the preparation presence. However, it seems that, unlike females, the lifespan extension in males was provided by lowering the age-dependent mortality.

In our understanding, the estimates *A* and *α* are not related precisely to the aging process itself, and tell us nothing about the rate of physiological changes in an individual. Nevertheless, these parameters could be examined as a good quantitative representation of the most susceptible age stratum in the cohort. For instance, a high *A* value would indicate accelerated dying among young individuals, while a high *α* would indicate quick dying in a group of older individuals, which, in our case, constitutes more than 90% of the whole cohort at the time of its half-life. Thus, in this case, we could suppose that the action of *R. rosea* might depend on the physiological state of the organism, accelerating the death of the least robust organisms, supporting survival of the organisms with a moderate robustness, and not influencing the most robust individuals. This approach partly explains why *R. rosea* supports survival of ‘young’ female *Drosophila*, suggesting that they are more resistant to environmental perturbations than males of the same age. It was also reported previously that females live longer and are more resistant to different stresses than male flies [46].

As a next step, we checked ‘life-quality’ indices, conventionally measured for *Drosophila* and other animal models of aging. Since *R. rosea* is known mainly by its adaptogenic properties, we also checked age-related changes in stress resistance of the treated flies.

### *R. rosea* supplementation increases climbing activity, heat, and oxidative stress resistance

**Figure 4:**
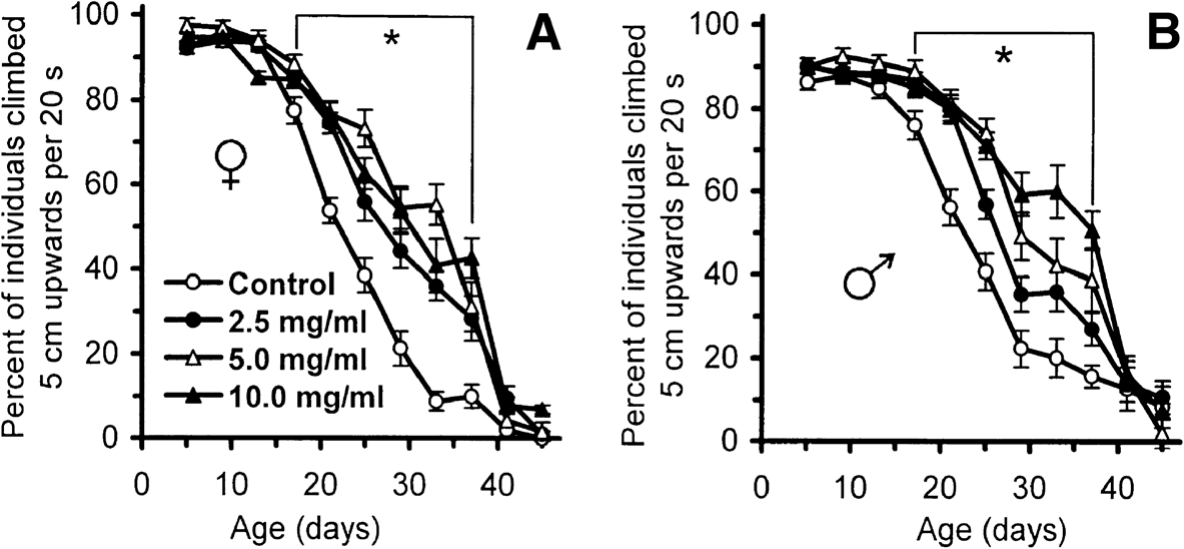
**Climbing activity of (A) female and (B) male flies fed by food with different concentrations of *R. rosea *rhizome.** Results are representative of two independent experiments. *Significantly different (*P* < 0.05) from control group as evaluated by Dunnett’s test.

Climbing activity is considered as a marker of healthspan in *Drosophila*[47]. The association of a decline in climbing activity with age has been established in *D. melanogaster*. We observed that *R. rosea* improved climbing activity of the flies over a wide concentration range. *Rhodiola-*fed flies showed significantly higher climbing activity from day 16 to day 40 compared with the controls. On day 33, 36-55% of females supplemented with 2.5 mg/ml, 5.0 mg/ml, and 10.0 mg/ml of *R. rosea* rhizome were able to pass 5 cm distance from food surface within 20 seconds, while among control flies only 10% could fulfill this task (Figure 4A, Table 2). Males showed similar results, with the strongest effect at 10.0 mg/ml *R. rosea* rhizome (Figure 4B, Table 2).

**Table 2:** **Induced climbing activity of the control flies and flies fed food supplemented with *R. rosea *** **rhizome**

**Sex**	***R. rosea *** **rhizome concentration (mg/ml)**	**Induced climbing activity at day 33 (%)**	**Induced climbing activity at day 45 (%)**
Females			
	0 (control)	9 ± 2	0
	2.5	36 ± 3*	1 ± 1
	5.0	55 ± 6*	2 ± 1
	10.0	41 ± 5*	7 ± 2
Males			
	0 (control)	20 ± 5	8 ± 5
	2.5	36 ± 5*	11 ± 4
	5.0	42 ± 6*	2 ± 2
	10.0	60 ± 7*	7 ± 4

*Significantly different (*P* < 0.05) from control group as evaluated by Dunnett’s test.

**Figure 5:**
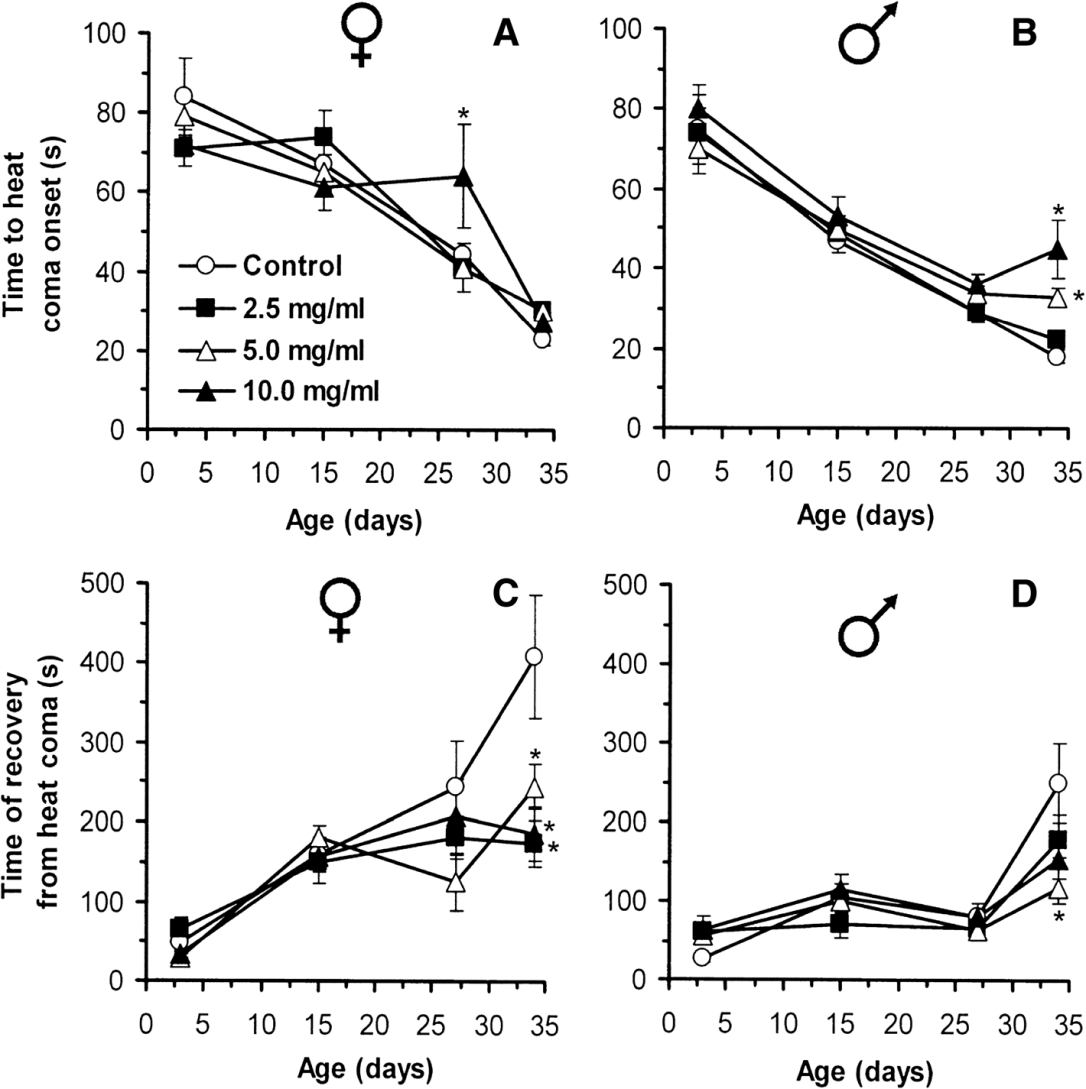
**Time of heat coma onset (A and B for females and males, respectively) and recovery (C and D for females and males, respectively) for flies fed food with different concentrations of *R. rosea *rhizome.** Results are representative of four independent experiments. *Significantly different (*P* < 0.05) from control group as evaluated by Dunnett’s test.

Many studies suggest a link between aging and stress resistance [19,20,48]. In this study, *R. rosea* rhizome conferred resistance to heat shock and menadione-induced stress. The newly enclosed and older (up to day 34) flies of both sexes did not demonstrate any difference between control and *Rhodiola*-fed groups in heat-induced coma onset (Figure 5). However, 34-day-old males, fed with the food supplemented with 5.0 mg/ml and 10.0 mg/ml *R. rosea* rhizome had a 1.8- and 2.5-fold longer recovery time, than controls, respectively (Figure 5B). Compared with controls, 34-day-old females supplemented with 2.5 mg/ml, 5.0 mg/ml, and 10.0 mg/ml of *R. rosea* rhizome recovered from heat coma 1.4-, 2.1-, and 1.6-fold faster, respectively (Figure 5C). Among males, only those receiving the 5.0 mg/ml *R. rosea* rhizome, recovered faster (2.6-times) from heat coma than the controls.

Recent studies have shown that heat-shock proteins (HSP) are involved in lifespan extension. In particular, *Rhodiola* preparations increased levels of HSP-16 in *C. elegans*[19]. Lifespan extension in *D. melanogaster* was also associated with enhanced Hsp22 levels [49,50]. It is known that Hsp22 is synthesized after a rapid heat shock [51] and may be important for cell survival under this stress. Kurapati and colleagues [49] showed that the level of Hsp22 mRNA was increased in both long- and short-lived lines between days 40 and 50, but later the expression of this gene was decreased. In this study, we used a rapid exposure to high temperature and observed that the flies fed the diet supplemented with *R. rosea* rhizome powder were more resistant to heat shock at the middle of the cohort lifetime.

**Figure 6:**
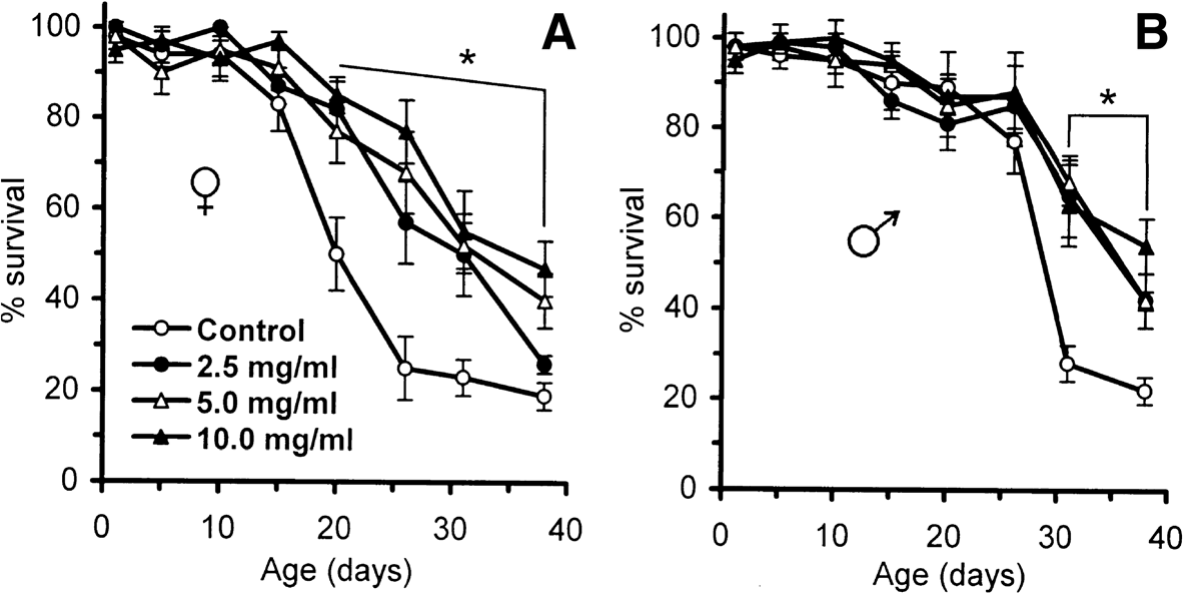
**Survival of (A) female and (B) male flies fed with different concentrations of *R. rosea *rhizome after exposure to 20 mM menadione.** Groups of flies of the indicated ages were taken from the cohorts and transferred to the vials with paper soaked in a solution containing 5% sucrose and 20 mM menadione sodium bisulfite. The percentage of survivors was determined after 24 h. Results are representative of four independent experiments. *Significantly different (*P* < 0.05) from control group as evaluated by Dunnett’s test.

Menadione (synthetic provitamin K) is a compound that produces superoxide-anion in a redox-cycling way [52]. In this study, an age-dependent change in menadione sensitivity was observed for both males and females (Figure 6). Among young flies (<16 days), no significant difference in sensitivity was observed between control and *Rhodiola*-fed groups exposed to 20 mM menadione, whereas older flies fed the herb preparation were more resistant. A 2.3-fold higher resistance was observed in 31-day-old males (Figure 6B) and a 2.4-fold higher resistance was observed in 20-day-old females (Figure 6A). The difference among treated groups (2.5 mg/ml, 5.0 mg/ml, and 10.0 mg/ml *R. rosea* rhizome) was not statistically significant.

It was previously shown that treatment of pond snail *Limnaea stagnalis* larvae by *R. rosea* extract conferred resistance to both 600 μM menadione and heat shock under 43°C [17]. Later, it was shown on *C. elegans* that adaptogens, including *R. rosea*, activate DAF-16/FOXO transcription factor, promoting change of its intracellular localization. In another study, performed on bladder cancer cell lines, *R. rosea* extract and salidroside itself increased phosphorylation of AMP-activated protein kinase, leading, most probably, to the activation of mTOR pathway [53]. It is known that both FOXO and TOR pathways are referred to stress resistance. In particular, one of the FOXO downstream targets is manganese-containing superoxide dismutase [54], which is responsible for elimination of mitochondrial superoxide and probably capable of providing a defense against redox-cycling agents, such as menadione or paraquat. However, it was found that *R. rosea* did not activate antioxidant responses in human osteosarcoma-derived diploid fibroblast and neuroblastoma cell lines [55]. In general, the findings on activation of HSP or antioxidant defenses by *R. rosea* are controversial. They imply that influence of *R. rosea* on signaling pathways is either transient, or that it does not operate directly with reactive oxygen species, but rather promotes more effective elimination of oxidized molecules (for example, autophagy).

### *R. rosea* supplementation enhances fly fecundity

Many conditions, mutations, and compounds that prolong lifespan may reduce reproduction [56]. From a medical and ethical point of view, lifespan extension at a cost of reduced reproduction would not be acceptable for the human population. In this context, there is a clear contemporary trend in searching for anti-aging remedies that do not affect reproduction [56]. This point seemed to us reasonable in the actual experiments. In previous studies, high doses of *R. rosea* powder or extract decreased egg-laying ability of fruit flies [7,8]. We checked the same for the preparation of *R. rosea*, collected in the Ukrainian Carpathian Mountains.

**Figure 7:**
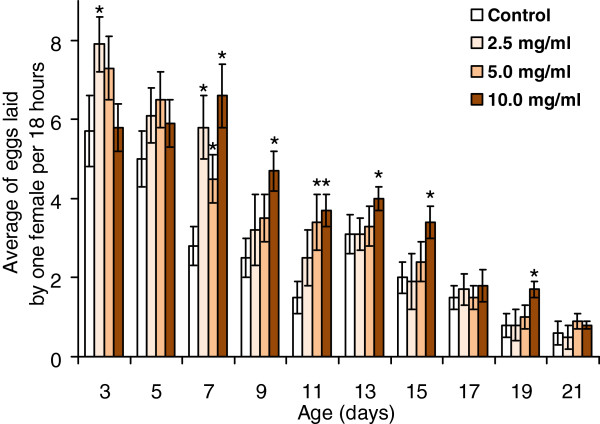
**Fecundity of flies fed with different concentrations of *R. rosea *rhizome supplement at different ages.** *Significantly different (*P* < 0.05) from control group as evaluated by Dunnett’s test.

A gradual decrease in fecundity with age in all treatment groups was observed, but *R. rosea* rhizome enhanced the fecundity in most age groups (Figure 7). The flies fed a diet supplemented with *R. rosea* rhizome demonstrated a higher fecundity on days 3 (2.5 mg/ml), 7 (all concentrations), 9 (10.0 mg/ml), 11 (all concentrations), 15, and 19 (10.0 mg/ml). It seems that *R. rosea* rhizome at 2.5 and 5.0 mg/ml provided a higher fecundity in young flies until approximately day 11, while 10.0 mg/ml of *R. rosea* rhizome enhanced fecundity from day 7 until day 19. The maximum effect was found at 10.0 mg/ml. There was found to be a positive relationship between fly fecundity and supplementation with 10.0 mg/ml *R. rosea* (the point-biserial correlation coefficient was equal 0.38 with *P* = 0.0033).

**Figure 8:**
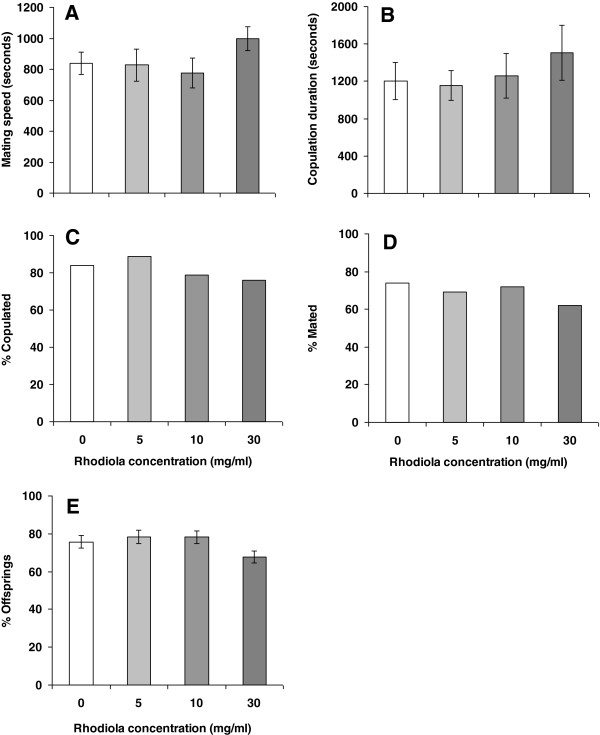
**Mating speed (A), copulation duration (B), percentages of copulated pairs (C) and mated females (D), and number of flies eclosed from eggs (E) for flies fed with control food or with food supplemented by 5 mg/ml *Rhodiola *rhizome.** Significance of difference between groups was assessed using Student’s *t* test.

To investigate the possible positive effect of *R. rosea* preparation on the egg-laying ability of female *Drosophila*, we measured the mating speed and duration of copulation in the control and *Rhodiola*-treated flies. These parameters, as well as the percentage of mated females and copulated flies, were not significantly different at any concentration of *Rhodiola* rhizome used (Figure 8). The number of eclosed offspring was also not affected.

A decrease in fecundity is often considered to be due to caloric restriction. Current data on fecundity suggest that *R. rosea* preparation might not mimic calorie restriction. All our observations on fecundity partially support the assumption that *R. rosea* may preserve biogenic amines from oxidation by inhibition of monoamine oxidase. In particular, it has recently been shown that octopamine is required for egg release from ovary [57], while serotonin is needed for male ejaculation [58].

### Effects of protein-to-carbohydrate ratio on the lifespan extension by *Rhodiola*

Recent studies have shown that bioactive compounds of *R. rosea*, particularly salidroside, may affect the mTOR (mammalian target of rapamycin) pathway [53]. It is known, that dietary protein-to-carbohydrate (P:C) ratio influences TOR signaling in *D. melanogaster*[29] and hence, lifespan, stress resistance, and food consumption [13]. In this way, a specific diet could modulate the lifespan-prolonging effect of *R. rosea*. In this study, we conducted a pilot experiment assessing lifespan extension by *R. rosea* on eight diets with different P:C ratios. The lifespan assays were conducted using mortality cages [59]. Yeast was used as the protein source, while sucrose was used as the carbohydrate source.

**Figure 9:**
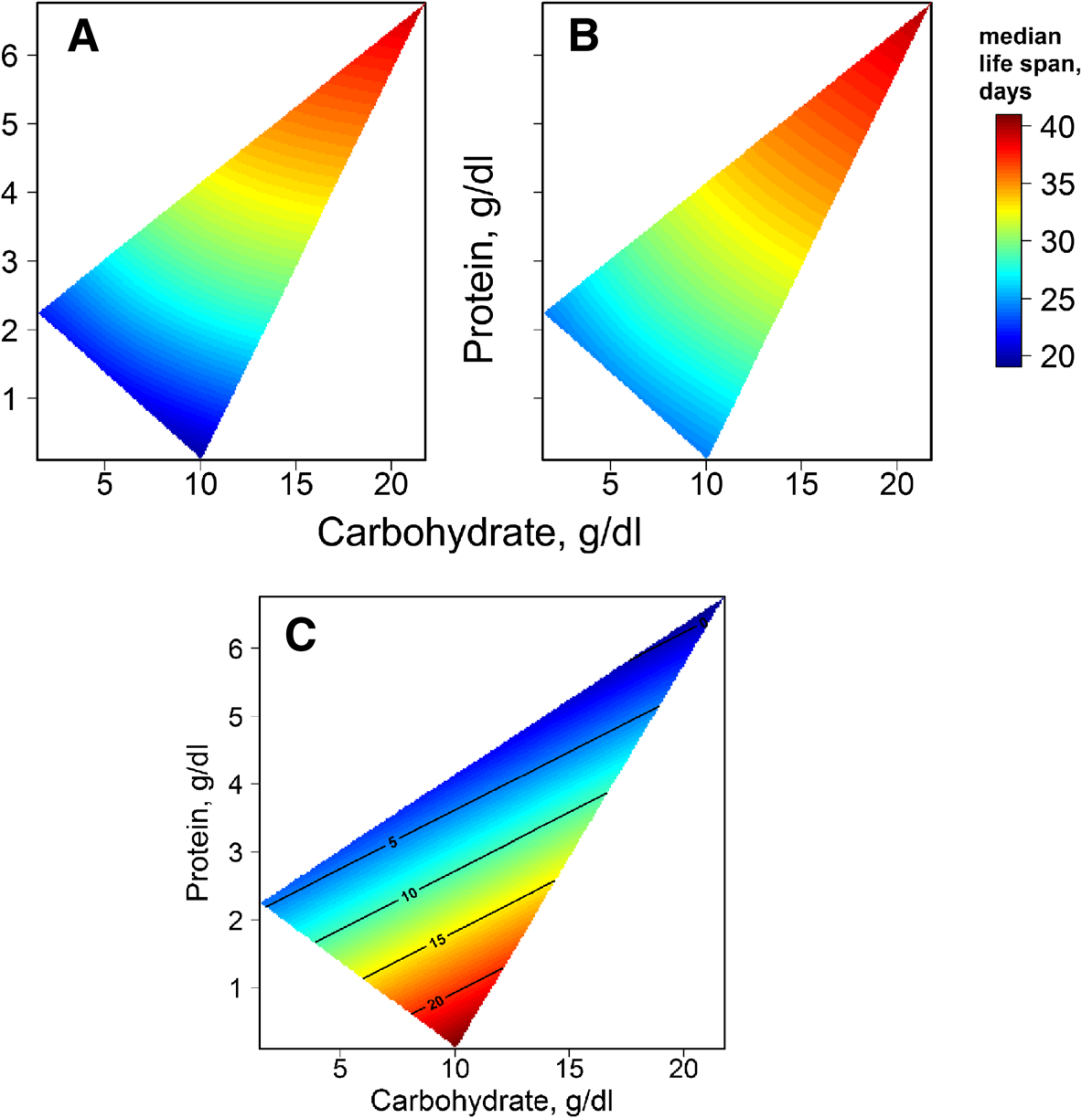
**Effect of protein-to-carbohydrate ratio (P:C) on lifespan extension by *R. rosea *rhizome preparation. A**, mean lifespan of females fed with control diets with different P:C ratios; **B**, mean lifespan of females fed diets with different P:C ratios and additionally supplemented with 5 mg/ml *R. rosea* rhizome powder; **C**, percent of lifespan extension by *R. rosea* on diets with different P:C ratios.

We obtained similar results on the diet with composition close to the one described previously. Diet-response surfaces show that the highest median lifespan was reached on diets with a P:C ratio around 0.36 for the diet with 5 g/l yeast and sucrose (Figure 9, A and B). These data converge with those described by Skorupa *et al*. [28]. However, the environment, namely the vials or mortality cage, as well as peculiarities of protein and micronutrient source, might also influence lifespan parameters. In particular, in our previous study, fruit flies lived approximately 80 days in vials on the diet with 10% sucrose with only 0.25% yeast extract [60], whereas in this study, fruit flies lived around 30 days in cages on a similar diet, (0.25% yeast, 10% sucrose; corresponding to around 10.1% carbohydrates and 0.11% protein).

Rhizome powder of *R. rosea* added to the diet at a concentration of 5 mg/ml extended mean lifespan by up to 11% to 15% on diets with P:C ratios of 0.044, 0.2, and 0.36, except the diet with 15% yeast and sucrose (15Y:15S), and up to 21% on the diet with P:C ratio 0.011 (Figure 9C). However, there was no significant extension on the diets with P:C ratios 1 (less than 9%) and 0.36 (15Y:15S), while on the diet with a P:C ratio 1.5 (5Y:0.25S) *R. rosea* supplementation decreased lifespan by about 10%.

These data lead us to suggest that the macronutrients, being at high concentration in the food, may cancel out the beneficial effect of *R. rosea* on *Drosophila* lifespan. This effect seems to be caused by the increased amount of protein in the diet. Additionally, the P:C ratio along with food consumption should also be taken into account. 

## Conclusions

Our current data indicate that *R. rosea* from the Carpathian Mountains increases lifespan and improves healthspan in a fruit fly line caught in the same region. However, the lifespan-prolonging effect of *R. rosea* on the fruit fly depends on the protein-to-carbohydrate ratio fed to it. Diets with high protein-to-carbohydrate ratios or high caloricity do not support the beneficial action of *R. rosea* on longevity. We also demonstrated that lifespan extension by *R. rosea* might depend on the physiological state of organism, being beneficial for individuals with moderate robustness. The maximum anti-stress effect of *R. rosea* is also observed for individuals with ages close to the half-life of the cohort. In addition to lifespan extension, *R. rosea* retards age-dependent decline in ‘life-quality’ indices of aged *D. melanogaster* individuals, such as climbing activity. The preparation from Carpathian *R. rosea* used in our study also has a positive effect on fruit fly egg-laying ability.

Although our work mainly supports previous reports on the anti-aging effects of *R. rosea* preparations, more work is needed to optimize dosage, evaluate its anti-aging effects on other model organisms, such as mammals, and identify putative and active compounds of *R. rosea* that might provide an opportunity to decipher the molecular mechanisms of *Rhodiola* components. Current data on these mechanisms remain controversial, suggesting involvement of the key regulators of longevity and stress resistance, namely transcription factor FOXO and TOR kinase. However, all molecular studies were performed on different models. We suppose that these mechanisms differ in unicellular and multicellular organisms, as well as in cell cultures, most of which are derived from tumors. The difference may be between simplistic organisms, like fruit flies or nematodes, and organisms with highly developed hormonal system, like mammals. Thus, future research foresees a molecular approach, beginning with unicellular organisms and purified bioactive compounds of *R. rosea*, and ending up with vertebrate models, close to human beings.

## Methods

### *Drosophila* stock and media

Founder flies (called ‘IF’) were collected in the southeast part of Ivano-Frankivsk (Western Ukraine) in August 2007. For the present investigations, the 20th and further generations from founder flies were used. General stocks were reared on a medium containing 5% molasses, 6% baker’s yeast and 1.3% agar-agar. For mold growth inhibition, the medium was supplemented by 0.4% propionic acid. Experimental food contained 5% sucrose and yeast and was solidified with 1.5% of agar. Rhizomes of *R. rosea* (collected in the Ukrainian Carpathian Mountains near Lazeshchyna) were finely ground and added to experimental food in concentrations of 2.5, 5.0, 10.0, and 30.0 mg/ml. Flies were reared at 25°C with a 12:12 light–dark cycle and a humidity of about 60%. All components of the fly food were taken from local manufacturers. Among them, propionic acid was of analytical grade.

### *Rhodiola rosea* preparation

Alkemists Pharmaceuticals (Costa Mesa, CA) characterized our *R. rosea* rhizome powder using high-performance liquid chromatography (HPLC). According to all animal and clinical studies, extracts of *R. rosea* should be standardized to greater than 1.5% salidroside and about 3% total rosavins (rosavin, rosarin, and rosin). The HPLC of our rhizome powder showed 1.9% salidroside and 2.5% total rosavins.

### Lifespan assay

Mated flies were separated and transferred into glass vials (15 × 150 mm) containing 1.25 ml of the experimental food. Each vial contained ten flies. Flies were transferred to vials with fresh food every other day and survivors were counted. Survival curves show means (by each time point) from three independent lifespan tests, in which about 150 to 400 flies of every sex for each concentration of *R. rosea* rhizome powder were used. Maximum lifespan was defined as the mean lifespan of the last 10% survivors. Survivorship curves were fitted by the equation:

$$N_{t}=N_{0}\cdot \exp\left\{\frac{A\left(1,-,\exp,\left(\mathrm{\alpha t}\right)\right)}{\alpha }\right\}$$

[61], where *N*_t_ is the number of live individuals at any time, *N*_0_ is the initial cohort size, *A* is the age-independent parameter of the Gompertz equation, and *α* is the age-dependent parameter of the Gompertz equation. R-project software (version 2.9.2) with the package *minpack.lm* was used for the calculation of Gompertz equation parameters from this function minimized by Levenberg-Marquardt method.

### Dietary response experiments

Diets with different concentrations (in g/dl) of yeast (Y) and sucrose (S) were used: 0.25Y:10S, 1Y:10S, 5Y:0.25S, 5Y:1S, 5Y:5S, 5Y:10S, 10Y:10S, 15Y:15S. Food was solidified with 1.5% agar and preserved from mold growth by 0.4% propionic acid. Cages were made from 15-cm diameter pipe. A plastic vial with food was screwed to the cage through a hole in the sidewall. The number of dead flies was checked every second day. Dead individuals were sucked out by an aspirator through the rubber-covered hole on the sidewall opposite to the food vial hole.

Diet-response surfaces were visualized by non-parametric thin splines using the *fields* package in R (version 2.14.1).

### Food consumption assay

Food consumption was measured as described by Lushchak *et al*. [62] with the following modifications. Groups of ten flies fed each particular diet were placed for 15 min on the same food with 0.5% food dye erioglaucine. After feeding, each group of flies was immediately frozen in liquid nitrogen. The flies were then homogenized in 100 μl of 50 mM potassium phosphate buffer (pH 7.5) and centrifuged at 13,500*g* for 15 min, and the supernatant was transferred to a new tube containing 100 μl of the buffer for dilution. Diluted samples were measured in a 96-well microplate reader at 629 nm. A solution of dye diluted in 50 mM potassium phosphate buffer was used to build a calibration curve. The optical density of the homogenates from flies consuming corresponding diets without the dye was used as a blank. Alternatively, fly feeding was quantified by direct observation, as described in [31].

### Physiology assays

To assess fruit fly fecundity, one male and one female fly were reared in small vials (15 × 60 mm) with 0.7 ml of experimental food with or without *R. rosea* rhizome supplement. Food was changed every other day. The number of eggs laid by one female was determined at 18 h after fly transfer on fresh food. Each bar on the histogram represents a mean ± standard error of the mean (SEM) from the values obtained from 25 fly pairs for each concentration of *R. rosea* rhizome.

The mating speed, copulation duration, percentage of copulated flies and number of mated females were determined by the protocol described by Chadha [63].

To test climbing activity, ten flies of respective age were gently tapped to the bottom of the vial. After 20 seconds, the percentage of flies that passed a distance of 5 cm was counted. Each point of the curve represents a mean ± SEM from 24 values collected at two independent measurements. The measurement was routinely conducted for 12 vials with eight to ten experimental flies per dose of *R. rosea* root powder, per sex. Each vial was tested three times at each time point and mean values were calculated.

To determine menadione resistance, ten flies were transferred every fifth or sixth day into empty vials for 4 h of starvation. After starvation, flies were transferred into vials containing folded and rammed strips (2.4 × 12 cm) of four-layer cellulose filter paper soaked with 0.8 ml of 20 mM menadione sodium bisulfite (Sigma-Aldrich Co.) in 5% sucrose solution. Survivors were counted after 24 h of exposure.

To evaluate resistance to heat stress, single flies were transferred into small glass vials with cotton stoppers. Vials were incubated in a water bath adjusted to 43°C. The time to onset of heat knock-down and the time to full restoration of mobility were recorded.

### Statistical analysis

Comparison of survivorship was performed by Wilcoxon rank sum test using R-project software with the package *exactRankTests*. Climbing activity, oxidative stress, and heat-shock resistance, as well as fecundity data were analyzed using Dunnett’s test. Data from all other assays were compared by Student’s *t*-test.

## Abbreviations

AMPK: AMP-activated protein kinase; FOXO: forkhead box O; HPLC: high-performance liquid chromatography; HSP: heat-shock proteins; mTOR: mammalian target of rapamycin; P:C: protein-to-carbohydrate ratio; SEM: standard error of the mean; TOR: target of rapamycin.

## Competing interests

The authors declare that they have no competing interests.

## Authors’ contributions

DVG performed the fecundity assay, participated in the lifespan, climbing activity, and menadione resistance assays, analyzed the Gompertz equation estimates, diet response surfaces and the fecundity and heat-shock resistance data, wrote the manuscript draft, and edited all its subsequent versions. ISY performed dietary response and food consumption assays. VIL co-designed research, critically discussed data, and co-edited the manuscript. MJ arranged HPLC analysis for rhizome of Carpathian *R. rosea*, made critical remarks on data performance, and co-edited the manuscript. OVL designed research, governed all assays, took part in lifespan, climbing activity, menadione and heat-shock resistance assays, analyzed lifespan, climbing activity, menadione resistance data, and co-edited the final version of the manuscript. All authors read and approved the final manuscript.

## Supplementary Material

Additional file 1: Figure S1 Feeding rates of female *Drosophila* on food media with different nutrient concentrations and supplemented with *R. rosea*. Feeding rates were recorded by direct observation as the proportion of time flies spent on the surface of the media with their proboscis extended and touching the food. Replicate measurements of the proportion of females feeding versus those not feeding were recorded during a 2-h period on the days shown. Significant differences were seen between flies fed the control diet (upper panel) and the diet supplemented with 5.0 mg/ml *R. rosea* (lower panel). Click here for file
