# Correction: Lifespan extension and delay of age-related functional decline caused by *Rhodiola rosea* depends on dietary macronutrient balance

**DOI:** 10.1186/2046-2395-2-12

**Published:** 2013-07-12

**Authors:** Dmytro V Gospodaryov, Ihor S Yurkevych, Oleh V Lushchak, Volodymyr I Lushchak

**Affiliations:** 1 Department of Biochemistry and Biotechnology, Vassyl Stefanyk Precarpathian National University, Ivano-Frankivsk 76025 Ukraine; 2 Department of Zoology, Stockholm University, S-10691 Stockholm, Sweden

## Correction

Following publication of the article entitled “Lifespan extension and delay of age-related functional decline caused by *Rhodiola rosea* depends on dietary macronutrient balance” [1] Mahtab Jafari requested to be removed from the author list and was not aware of the manuscript’s submission to *Longevity* & *Healthspan*. Mahtab Jafari has been removed from the corrected author list above and all authors also agreed to the revised order of the corrected author list.

The authors would like to thank Mahtab Jafari for her help in preparing an earlier version of the manuscript and the arrangement of HPLC analysis for rhizome of Carpathian *Rhodiola rosea*.
