# Supplementary figures and images for: Lifespan extension and delay of age-related functional decline caused by Rhodiola rosea depends on dietary macronutrient balance
Source: Longev Healthspan. 2013 Apr 2;2:5. doi: 10.1186/2046-2395-2-5 (PMC3922952; doi:10.1186/2046-2395-2-5)

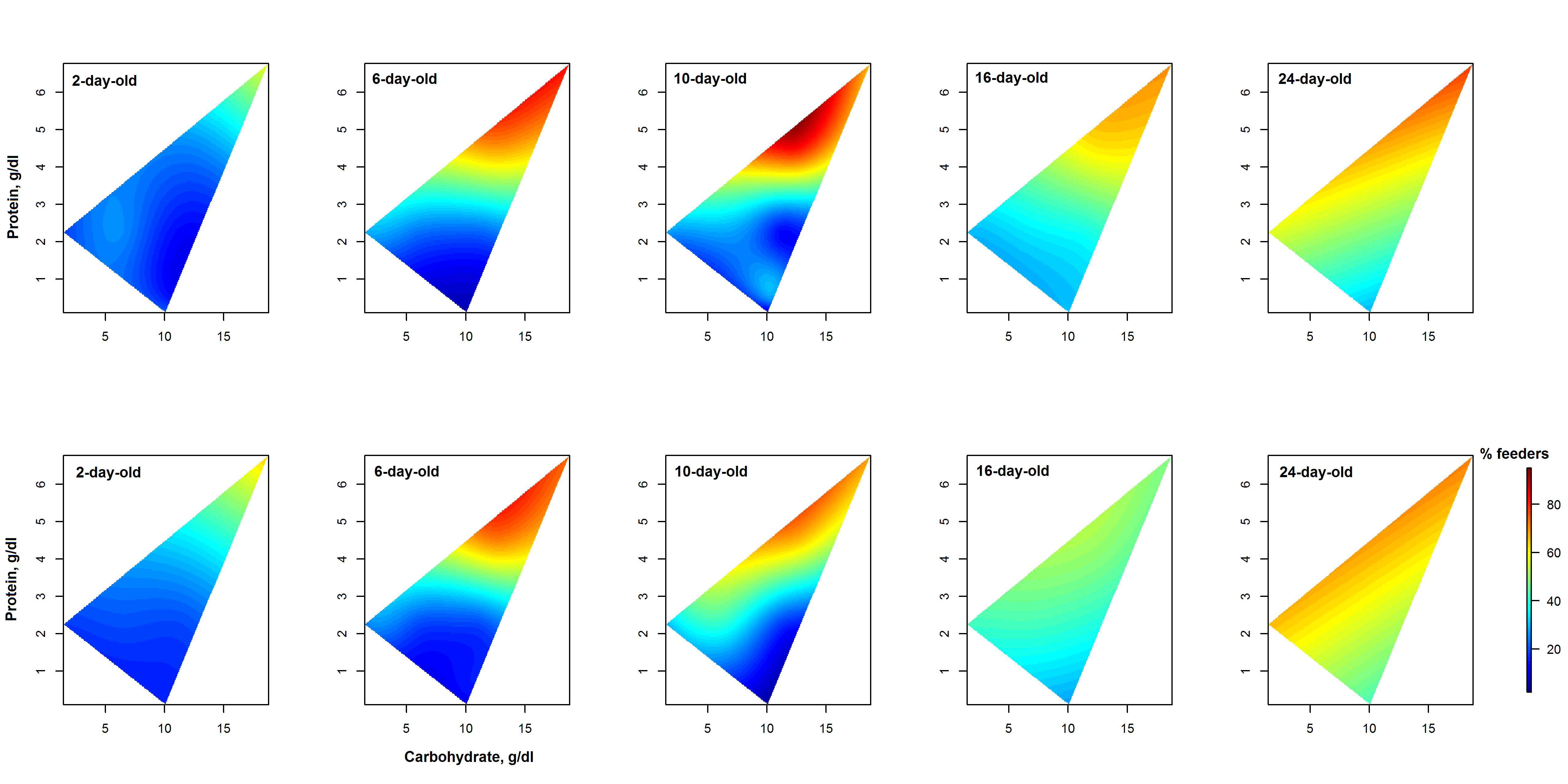

Supplement: Additional file 1: Figure S1 — Feeding rates of female Drosophila on food media with different nutrient concentrations and supplemented with R. rosea. Feeding rates were recorded by direct observation as the proportion of time flies spent on the surface of the media with their proboscis extended and touching the food. Replicate measurements of the proportion of females feeding versus those not feeding were recorded during a 2-h period on the days shown. Significant differences were seen between flies fed the control diet (upper panel) and the diet supplemented with 5.0 mg/ml R. rosea (lower panel). [file 2046-2395-2-5-S1.tiff]
